# *Besnoitia besnoiti–*driven endothelial host cell cycle alteration

**DOI:** 10.1007/s00436-020-06744-x

**Published:** 2020-06-17

**Authors:** Zahady D. Velásquez, Sara Lopez-Osorio, Learta Pervizaj-Oruqaj, Susanne Herold, Carlos Hermosilla, Anja Taubert

**Affiliations:** 1 Institute of Parasitology, Biomedical Research Center Seltersberg, Justus Liebig University, Schubertstr. 81, 35392 Giessen, Germany; 2 Research Group CIVAB, School of Veterinary Medicine, Faculty of Agrarian Sciences, University of Antioquia, Medellín, Colombia; 3 Cardio Pulmonary Institute (CPI), Giessen, Germany; 4 Universities Giessen & Marburg Lung Center (UGMLC), Giessen, Germany; 5 German Center for Lung Research (DZL), Giessen, Germany

**Keywords:** *Besnoitia besnoiti*, Apicomplexan parasites, Coccidia, Cell cycle arrest, Histone H3 S10

## Abstract

*Besnoitia besnoiti* is an important obligate intracellular parasite of cattle which primarily infects host endothelial cells of blood vessels during the acute phase of infection. Similar to the closely related parasite *Toxoplasma gondii*, *B. besnoiti* has fast proliferating properties leading to rapid host cell lysis within 24–30 h p.i. in vitro. Some apicomplexan parasites were demonstrated to modulate the host cellular cell cycle to successfully perform their intracellular development. As such, we recently demonstrated that *T. gondii* tachyzoites induce G2/M arrest accompanied by chromosome missegregation, cell spindle alteration, formation of supernumerary centrosomes, and cytokinesis impairment when infecting primary bovine umbilical vein endothelial cells (BUVEC). Here, we follow a comparative approach by using the same host endothelial cell system for *B. besnoiti* infections. The current data showed that—in terms of host cell cycle modulation—infections of BUVEC by *B. besnoiti* tachyzoites indeed differ significantly from those by *T. gondii*. As such, cyclin expression patterns demonstrated a significant upregulation of cyclin E1 in *B. besnoiti–*infected BUVEC, thereby indicating parasite-driven host cell stasis at G1-to-S phase transition. In line, the mitotic phase of host cell cycle was not influenced since alterations of chromosome segregation, mitotic spindle formation, and cytokinesis were not observed. In contrast to respective *T. gondii*–related data, we furthermore found a significant upregulation of histone H3 (S10) phosphorylation in *B. besnoiti*–infected BUVEC, thereby indicating enhanced chromosome condensation to occur in these cells. In line to altered G1/S-transition, we here additionally showed that subcellular abundance of proliferating cell nuclear antigen (PCNA), a marker for G1 and S phase sub-stages, was affected by *B. besnoiti* since infected cells showed increased nuclear PCNA levels when compared with that of control cells.

## Introduction

The apicomplexan obligate intracellular protozoa *Besnoitia besnoiti* represents a coccidian parasite of major importance in cattle industry. *B. besnoiti* infection was classified as an “emerging disease” in Europe by the European Food Safety Authority in 2010 (EFSA). Bovine besnoitiosis leads to severe skin alterations, vulvitis, vaginitis, orchitis, and infertility of bulls and cows among other signs (Gollnick et al. 2018). Consequently, this parasite causes significant losses in commercial cattle industry and impairs individual animal welfare (Dubey and Lindsay 1996; Dubey 2003; Cortes et al. 2014).

It is well-known that apicomplexan parasites significantly modulate their host cells to guarantee successful intracellular development and proliferation. As such, they influence numerous host cellular pathways, such as apoptosis, autophagy, cytoskeleton, metabolism, or immune reactions. In this context, some reports have indicated that tachyzoites of *T. gondii* dysregulate the host cellular cell cycle (Brunet et al. 2008; Molestina et al. 2008; Velásquez et al. 2019). The cell cycle of mammalian cells represents a highly regulated and complex processes that includes successive progression of distinct cell cycle phases (G0-G1; S, and G2-M), which finally leads to cell division via cytokinesis. The cell cycle begins with the G1-phase (Gap-phase 1). In this step, the cell synthetizes mRNA and proteins that the next cell cycle steps. Afterward, the cell triggers the DNA synthesis machinery to duplicate its complete genome, in the so-called S-phase. Once this process is completed, the cell enters into a new process of growing and synthetizing proteins, called the G2-phase. Finally, the cell activates the genome division process, called mitosis, which will give rise to two daughter cells with the same genome composition and size (M-phase and cytokinesis). The transition to each phase is tightly regulated by specific checkpoint proteins and is based on sequential activation or inactivation of cyclins, cyclin-dependent kinases (Cdk), and cyclin-dependent kinase inhibitors (CDK-inhibitor). For instance, G1-phase is regulated by D- and E-type cyclins, while S-phase is controlled by A-type cyclins and G2/M-phase A-type and B-type cyclins (Vermeulen et al. 2003). Cyclin and its CDK partner modulates an intracellular signal that allows for the cell cycle progression. On the contrary, CDK-inhibitors regulate the cyclins-CDK complex activity and/or degradation to allow the correct cell cycle development.

In case of protozoan infections, data indicate a species-specific host cellular cell cycle dysregulation. As such, *T. gondii* and *Leishmania* spp. induce cell cycle arrest and eventually dampen host cell proliferation (Brunet et al. 2008; Costales et al. 2009; Kim et al. 2016; Kuzmenok et al. 2005; Molestina et al. 2008; Scanlon et al. 2000; Velásquez et al. 2019), while *Theileria annulata* and *Theileria parva* trigger host cell division and proliferation (von Schubert et al. 2010; Wiens et al. 2014) and induce segregation of *Theileria* merozoites to each developing daughter cell. Conversely, *L. amazonensis* interferes early in cell cycle by G0/G1-phase arrest (Kuzmenok et al. 2005). In contrast, *Plasmodium falciparum* infections of HepG2 cells affect mitosis and lead to a binucleated phenotype and a lack of cell division (Hanson et al. 2015). In the case of *T. gondii*, available data record different modes of action, on one side, an infection-driven shift from G0/G1 to S phase with an accumulation of host cells in S phase (Molestina et al. 2008; Lavine and Arrizabalaga 2009). On the other side, a host cellular arrest in the G2 phase (Brunet et al. 2008) or even both (Kim et al. 2016), thereby most probably reflecting cell type–specific reactions. We recently reported that *T. gondii* infections of primary bovine umbilical vein endothelial cells (BUVEC) lead to a G2/M arrest and trigger severe defects during mitosis as propagated by chromosome missegregation, supernumerary centrosome formation, and cytokinesis impairment (Velásquez et al. 2019). Given that no data exist on *B. besnoiti*–triggered host cell cycle modulation, and also to generate comparative data with *T. gondii*, we here used the same type (BUVEC) for *B. besnoiti* infections in order to replicate in vivo infections as closely as possible and analyzed the impact of this obligate intracellular parasite on cell cycle progression. We here show for the first time that *B. besnoiti* infection indeed alters cell cycle–related molecules (e.g., cyclin E1, p27-kip1) but differs in its effects from *T. gondii*, therefore modulating host endothelial cell cycle progression in a parasite species-specific manner.

## Material and methods

### Primary bovine umbilical vein endothelial cell isolation and maintenance

Primary bovine umbilical vein endothelial cells (BUVEC) were isolated from umbilical veins obtained from calves born by *sectio caesarea* at the Justus Liebig University Giessen. Therefore, umbilical cords were kept at 4 °C in 0.9% HBSS–HEPES buffer (pH 7.4; Gibco, Grand Island, NY, USA) supplemented with 1% penicillin (500 U/ml; Sigma, St. Louis, MO, USA) and streptomycin (500 μg/ml; Sigma) for a maximum of 16 h before use. For the isolation of endothelial cells, 0.025% collagenase type II (Worthington Biochemical Corporation) suspended in Pucks solution (Gibco) was infused into the lumen of ligated umbilical veins and incubated for 20 min at 37 °C in 5% CO_2_ atmosphere. After gently massaging the umbilical veins, the cell suspension was collected in cell culture medium and supplemented with 1 ml fetal calf serum (FCS, Gibco) in order to inactivate collagenase. After two washes (350×*g*, 12 min, RT), cells were resuspended in complete endothelial cell growth medium (ECGM, PromoCell, supplemented with 10% FCS), plated in 25-cm^2^ tissue plastic culture flasks (Greiner) and kept at 37 °C in 5% CO_2_ atmosphere. BUVEC were cultured in modified ECGM medium (EGCM, diluted at 30% in M199 medium, supplemented with 5% FCS (Greiner) and 1% penicillin and streptomycin) with medium changes every 2–3 days. BUVEC cell layers were used for infection after 3 passages in vitro. All bovine primary endothelial cells samples were conducted in accordance with the Institutional Ethics Commission of Justus Liebig Universität of Gießen (Germany), and in accordance with the current European Animal Welfare Legislation: ART13TFEU.

### Parasite tachyzoite maintenance

Tachyzoites of *Besnoitia besnoiti* (strain Bb1Evora03) were maintained by serial passages either in primary bovine umbilical vein endothelial cells (BUVEC) or African green monkey kidney epithelial cells (MARC-145) according to (Muñoz Caro et al. 2014). Confluent BUVEC monolayers in 25-cm^2^ flasks were infected with 2.5 × 10^5^ freshly isolated *B. besnoiti* tachyzoites. From 48 h p.i. onwards, free-released viable tachyzoites were collected from BUVEC culture supernatants, filtered through a 5-μm syringe filter (Sartorius AG), washed in modECGM and pelleted (400×*g*, 12 min). Tachyzoites were counted in a Neubauer chamber and suspended in modECGM until further experimental use.

### Protein extraction

Proteins from infected and non-infected BUVEC were extracted by cell sonication (20 s, 5 times) in RIPA buffer (50 mM Tris-HCl, pH 7.4; 1% NP-40; 0.5% Na-deoxycholate; 0.1% SDS; 150 mM NaCl; 2 mM EDTA; 50 mM NaF, all Roth) supplemented with a protease inhibitor cocktail (Sigma-Aldrich). Cell homogenates were centrifuged (10,000×*g*, 10 min, 4 °C) to sediment intact cells and nuclei. The protein content of RIPA buffer-soluble cell supernatant was quantified via Coomassie Plus (Bradford) Assay Kit (Thermo Scientific) following the manufacturer’s instructions. Same protocol was applied for the tachyzoites protein extract production.

### Cell nuclei extraction

Four BUVEC isolates were seed in T-25 flasks and infected with *B. besnoiti* (MOI 1:5). For controls, non-infected cells were used. After 24 h p.i., cells were detached by a cell scraper (Thermo Fisher Scientific) and collected in 500 μL fractionation buffer (20 mM HEPES, pH 7.4, 10 mM KCL, 2 mM MgCl_2_, 1 mM EDTA, 1 mM EGTA, 1 mM DTT, and protease inhibitor cocktail, Thermo Fisher Scientific). Cells were incubated for 15 min on ice, passed 10 times through a 27-gauge needle and kept on ice for 20 min. To separate nuclear and cytoplasmic fractions, samples were centrifuged at 720*×g* for 5 min. The pellet (nuclear fraction) was washed thrice in 500 μl fractionation buffer, and thereafter, nuclei were disrupted by passing through a 25-gauge needle (10x). Nuclear proteins were then obtained by centrifugation at 700*×g* for 10 min.

### SDS-PAGE and immunoblotting

For immunoblotting, samples were supplemented with 6 M urea. After boiling (95 °C, 5 min), total proteins (60 μg/slot) were separated in polyacrylamide gels (12% or 15%) via electrophoresis (100 V, 1.5 h; *tetra* system, BioRad). Proteins were transferred to polyvinylidene difluoride (PVDF) membranes (Millipore) (300 mA, 2 h). Blots were blocked in 3% BSA in TBS (50 mM Tris-Cl, pH 7.6; 150 mM NaCl containing 0.1% Tween (blocking solution); Sigma-Aldrich) for 1 h at RT and then incubated in primary antibodies (see Table 1) diluted in a blocking solution (overnight, 4 °C). Detection of vinculin was used as loading control for normalization of samples. Following three washings in TBS-Tween (0.1%) buffer, blots were incubated in adequate secondary antibody solutions (diluted in blocking solution, for dilution: see Table 1; 30 min, RT). Following three further washings in TBS-Tween buffer, signal detection was accomplished by an enhanced chemiluminescence detection system (ECL® plus kit, GE Healthcare) and recorded using a ChemoCam Imager (Intas Science Imaging). Protein masses were controlled by a protein ladder (PageRuler Plus® Prestained Protein Ladder ~ 10–250 kDa, Thermo Fisher Scientific). Protein band intensity quantification was analyzed using a Fiji Gel Analyzer® plugin. For all protein analyzed, the parasitic homologous protein was detected (data not shown).

**Table 1:** Primary antibodies used for western blot and immunofluorescence

Primary antibodies				
Antigen	Company	Cat. number	Origin	Dilution
Vinculin	Santa Cruz	sc-73614	Mouse	1:1000
Cyclin A2	Abcam	ab39	Mouse	1:1000
Cyclin B1	Abcam	ab32053	Rabbit	1:3000
Cyclin B1 S126	Abcam	ab133439	Goat	1:1000
Cyclin E1	Abcam	ab133266	Rabbit	1:1000
Histone H3 S10	Abcam	ab5176	Rabbit	1:1000
PCNA	Abcam	ab18197	Rabbit	1:1000
α-Tubulin	Thermo Fisher	A11126	Mouse	1:100

### Immunofluorescence assays

Cell layers were fixed with paraformaldehyde (4%, 15 min, RT), washed thrice with PBS and incubated in blocking/permeabilization solution (PBS with 3% BSA, 0.1% saponin; 1 h, RT). Thereafter, samples were incubated in primary antibodies (see Table 1) diluted in blocking/permeabilization solution (overnight, 4 °C, in a humidified chamber). After three washings in PBS, samples were submitted to secondary antibody solution (see Table 2; 30 min, RT, darkness). Cell nuclei were labeled with 4′,6-diamidin-2-phenylindol (DAPI) being present in mounting medium (Fluoromount G, ThermoFisher, 495952).

**Table 2:** Secondary antibodies used for western blot and immunofluorescence

Secondary antibodies				
Name	Company	Cat. number	Reactivity	Dilution
Goat anti-mouse IgG Peroxidase conjugated	Pierce	31430	Mouse	1:40000
Goat anti-rabbit IgG peroxidase conjugated	Pierce	31460	Rabbit	1:40000
AlexaFluor 488	Thermo Fisher	A11008	Rabbit	1:500
AlexaFluor 488	Thermo Fisher	A11001	Mouse	1:500
AlexaFluor 594	Thermo Fisher	R37117	Rabbit	1:500
AlexaFluor 594	Thermo Fisher	A11005	Mouse	1:500

### Flow cytometry–based analysis of cell cycle phases

Cellular DNA content was measured using the FxCycle Far® red stain reagent (Invitrogen, F10348) according to the manufacturer’s instructions. The samples were analyzed with a FACSCalibur® Analyzer (Becton-Dickinson, Heidelberg, Germany) applying 633/5 nm excitation and emission collected in a 660/20 bandpass. Cells were gated according to their size and granularity. Exclusively morphologically intact cells were included in the analysis. Data analysis was performed by the use of the FlowJo® (version 10.5.0) flow cytometry analysis software (FlowJo LLC, Ashland, OR).

### Confocal microscopy

All immunofluorescence analyses were performed by confocal microscopy (× 63 magnification with a numerical aperture of 1.4, LSM 710, Olympus). Two types of image acquisition were used: (i) multi-channel images which were merged to define the co-localization of the signal, and (ii) Z-stacks of 0.3–0.5 μm for cell spindle and chromosome detection. Image processing was carried out by Fiji ImageJ® using Z-projection and merged-channel-plugins being restricted to overall adjustment of brightness and contrast.

### Live cell 3D-holotomographic microscopy

3D-holotomographic images were obtained by using 3D Cell Explorer microscope (Nanolive® 3D) equipped with an × 60 magnification optic (*λ* = 520 nm, sample exposure 0.2 mW/mm2) and a field depth of 30 μm. Images were analyzed using the STEVE® software (Nanolive) to obtain a refractive index-based z-stack. Digital staining was applied according to the refractive index of intracellular structures. Nuclei were stained by DRAQ5 probe for vital DNA staining following the manufacturer instructions (DRAQ5™ Fluorescent Probe Solution, ThermoFischer).

### Statistical analysis

The data from the total cell number in the monolayer and for WB assay were expressed as mean ± SEM from six BUVEC isolates. For cell number– and FACS-based assays, one-way analysis of variance (non-parametric ANOVA) with Kruskal-Wallis post-test was performed using the GraphPad Prism® 7 software applying a significance level of 5%. For immunoblot-based analyses, unpaired two-tailed *T* tests were performed with Mann-Whitney post-test comparing controls vs infected cells, with a 95% confidence interval.

## Results

### *B. besnoiti* infections do not affect host cell proliferation

**Fig. 1:**
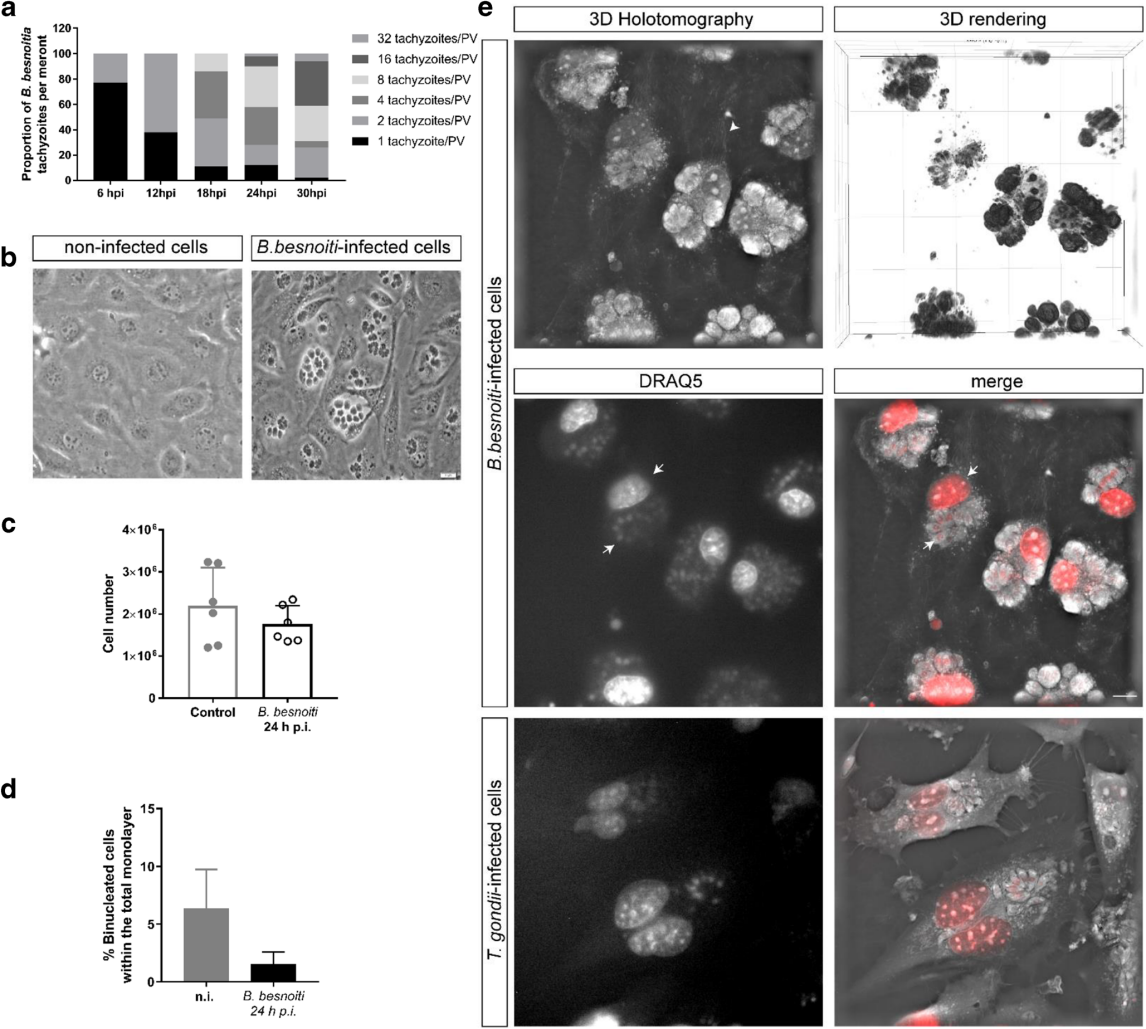
Infection of BUVEC with *B. besnoiti* tachyzoites and effect of infection on host cell proliferation. Sub-confluent BUVEC were infected with *B. besnoiti* tachyzoites at an MOI 5:1 and analyzed after 24 hpi. **a** Quantification of tachyzoites numbers/parasitophorous vacuole during 30 h of infection. The mature structures were observed between 24 and 30 h p.i. After this time point, all parasites were released. **b** Illustration of non-infected control cells and *B. besnoiti–*infected BUVEC at 24 h p.i. **c** Host cell proliferation analyzed by estimating cell numbers of *B. besnoiti*–infected BUVEC and non-infected controls at 24 h p.i. No statistically significant differences were observed, but it was a hint of decreased number of cells at the *B. besnoitia*–infected monolayer. **d** Quantification of binucleated host cells in *B. besnoiti*–infected BUVEC and non-infected controls. **e** Illustration of DRAQ5 (vital DNA staining, red)-stained host cell nuclei in *B. besnoiti*– and *T. gondii*–infected BUVEC (24 h p.i.) via 3D holotomographic microscopy (note: binucleated phenotype in case of *T. gondii*–infected BUVEC)

Even though *B. besnoiti* tachyzoites were recorded to successfully replicate in various immortalized host cell types (Cortes et al. 2007; Samish et al. 1988; Shkap et al. 1991), the kinetic of parasite division may differ depending on the host cell types infected in vivo. For this reason, we first estimated *B. besnoiti* division cycles in primary BUVEC by analyzing the number of tachyzoites present in each parasitophorous vacuole (PV) at a different time of infection (Fig. 1a). Using an MOI of 5:1 in three BUVEC isolates, we achieved an infection rate of 46.23 ± 14.73%. Overall, we observed a non-synchronous proliferation of tachyzoites in BUVEC. First, tachyzoite division (detection of 2-mers) was observed at 6 h p.i.; from there onwards, an ongoing division was estimated and most tachyzoites had divided at 18 h p.i. (Fig. 1a). At 24 h p.i., 1- to 32-mers were found in intracellular PVs (Fig. 1b).

The effect of *B. besnoiti* infections on host cell proliferation was examined by counting total BUVEC numbers. Given that we worked with a primary cell type, considerable variations in the size of cells and the time of cell division can be expected between isolates. Taking this into account, we included six biological replicates and used identical cell numbers for seeding. Given that *B. besnoiti* belongs to fast-replicating coccidian parasites and host cell lysis mainly occurs from 24 h p.i. onwards (thereby potentially inducing artifacts in host cell enumeration), we here restricted all host cell proliferation–based analyses to 1 day p.i. The determination of total host cell numbers revealed no statistically significant influence of *B. besnoiti* infections on host cell proliferation when compared with non-infected BUVEC controls (Fig. 1b–c). Nevertheless, the analysis of each individual BUVEC isolate after 24 h p.i., which showed a decreasing tendency in the number of cells in the monolayer (Fig. 1c**)**. This evidence was considered important because all samples, were seeded the same number and at the same time. Overall, four isolates from a total of six showed a decrease of the total amount of cells in the monolayer after 24 h p.i. Inter-donor variation in different parameters between BUVEC isolates in different experimental set-ups had been observed before, and, thus, we made efforts to interpret the variation on a case by case basis (Conejeros et al. 2019; Taubert et al. 2016).

We recently showed that *T. gondii* infections resulted in a significantly enhanced proportion of BUVEC presenting more than one nucleus (mainly binucleated BUVEC; Velásquez et al. 2019). For comparative reasons, we here also estimated the cell nucleus numbers in *B. besnoiti*–infected BUVEC and non-infected controls. Our results showed that *B. besnoiti* infections had no effect on host cell nucleus numbers (Fig. 1d). This was also tested via DRAQ5 (vital staining of nuclei)-based live cell 3D holotomographic microscopy (3D Nanolive®). By this novel live cell imaging technique, we showed that *B. besnoiti* infections did not affect the nuclear phenotype, while *T. gondii* infections, which were used for comparative reasons, indeed induced a binucleated phenotype in BUVEC (Fig. 1e).

### *B. besnoiti* induces host cell stasis at G1-to-S-phase transition

**Fig. 2:**
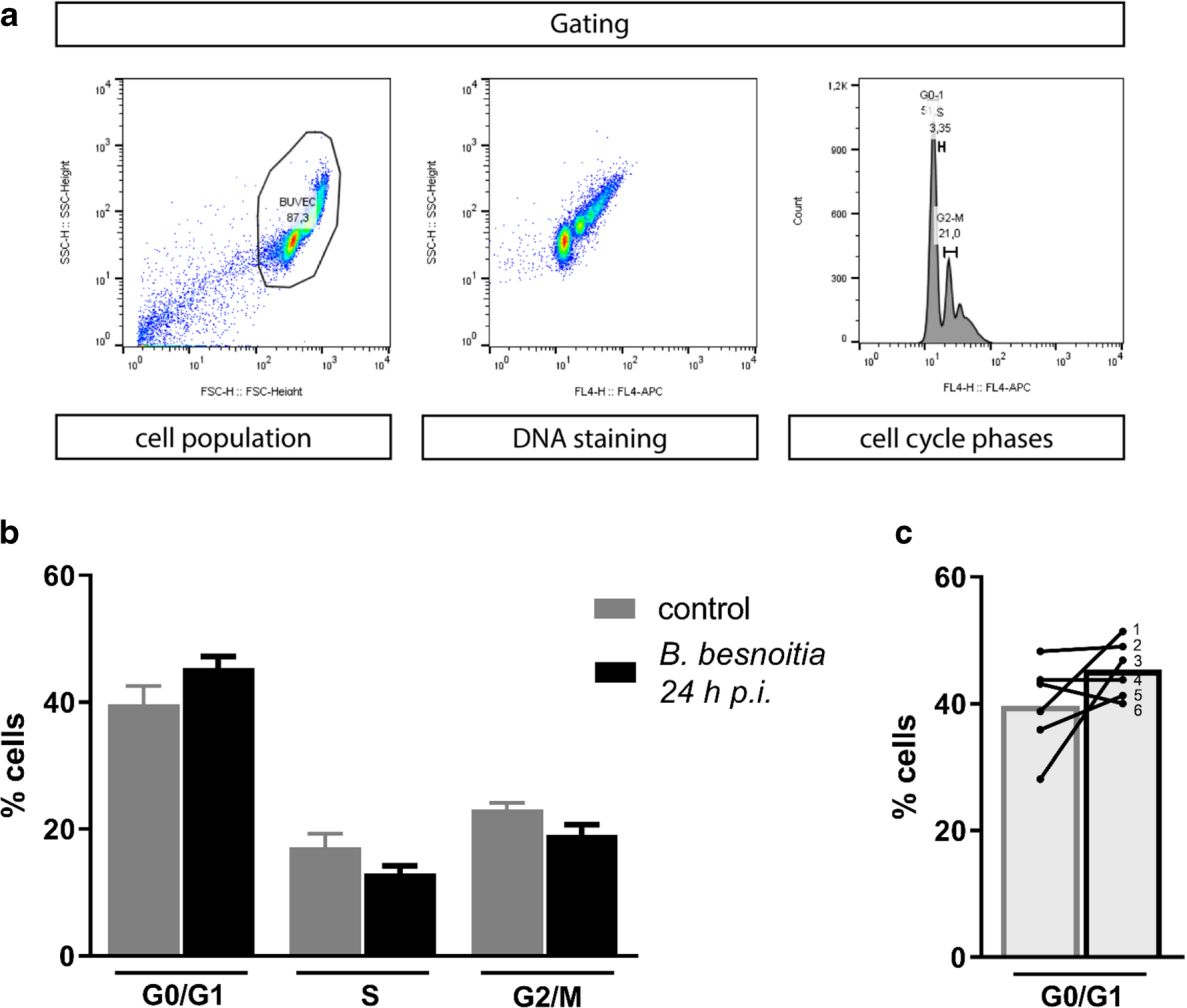
Distribution of cell cycle phases in *B. besnoiti*–infected BUVEC. BUVEC were infected with *B. besnoiti* and examined for DNA content at 24 h p.i. by FACS-based analyses. **a** Flowchart of the FACS analysis showing the total number of cells in G- (one genomic DNA copy), G2- (two genomic copies), and S- (the cell population in between both phases) phase. The cells were first gated to eliminate debris from the analysis. Furthermore, the DNA channel vs the population histograme was used to get the total number of cells in each peak. **b** Mean data obtained from *B. besnoiti–*infected BUVEC (*n* = 6), plotted as a percentage of the total cells vs DNA amount. Bars represent the median ± SD. **c** G1-phase graph from (b) comparing the BUVEC isolates distribution between non-infected and B. besnoiti-infected cells. Light increase in the number of cells was observed in the peak corresponding to G0/G1; however, the value was not statistically significant

**Fig. 3:**
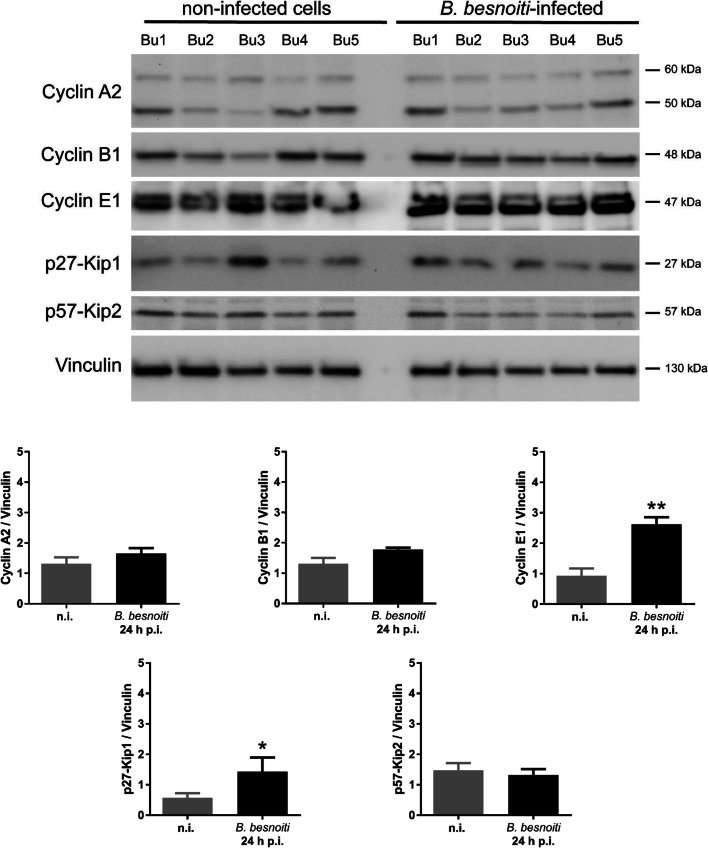
Analysis of cell cycle–related molecule expression in *B. besnoitia*–infected BUVEC. **a** Five biological replicates of BUVEC were infected with *B. besnoiti* tachyzoites and analyzed by western blotting for the abundance of the cell cycle–related molecules cyclin A2, cyclin B1, cyclin E, p27-Kip1, and p57-Kip2 at 24 h p.i. The density of the protein signals was quantified, and graphed as a relative ratio to vinculin (housekeeping protein). Cyclin E1 and p27-kip1 were upregulated in infected cells thereby indicating a G0/G1 cell cycle arrest. Bars represent the median ± SD of five biological replicates

To estimate whether *B. besnoiti* infections dysregulate host cellular cell cycle progression in BUVEC, we performed flow cytometry–based analyses on the cellular DNA content. This well-established method simply measures cellular DNA abundance, thereby allowing for the discrimination of the three main periods of the cell cycle (G0/G1-; S-; G2/M-phase). Even though this technique lacks high sensitivity, we could recently demonstrate a *T. gondii*–driven shift of cell cycle phases in BUVEC by applying this method (Velásquez et al. 2019). Here, the analysis was performed on BUVEC population in the SSC-H vs FSC-H graph (exemplary illustration in Fig. 2a), where we selected the three main peaks representing the three main phases of the cell cycle (G0/G1-; S-; G2/M-phase). However, by comparing total cell layers of *B. besnoiti*–infected BUVEC with non-infected ones, we could not identify any influence of *B. besnoiti* infections on host cell cycle progress (Fig. 2b). Nevertheless, the G1-phase of infected cells seemed to be higher in comparison with the non-infected monolayer. On account of this, we analyzed G0/G1-phase in more detail (Fig. 2c).We conducted a correlation between the value of each BUVEC isolate in the non-infected condition in comparison with the value of the same isolate, but in the *B. besnoitia–*infected layer. The results showed that 4/6 isolates revealed an increased number of cells in the G0/G1-phase (isolates 1–3, 5), 1/6 was unchanged (isolate 4), and 1/6 showed a decrease in the percentage of cells in G0/G1 (isolate 6). Given that analyses based on rough cellular DNA content may not be sensitive enough to detect minor changes in cell cycle progression, we additionally controlled *B. besnoiti*–infected BUVEC for protein abundance of several key molecules regulating cell cycle phases. Thus, cyclin B1 (indicative for G2 to M-phase transition), cyclin E1 (indicative for G1/S phase transition), and cyclin A2 (indicative for S/G2-phases transition) were controlled for expression via western blotting. Furthermore, p27-kip1 and p57-kip2 (inhibitors of G1-S transition cyclins) were analyzed in their abundance to control for a potential cell cycle arrest. Given that primary BUVEC monolayers vary considerably in their individual reactions, we here included five different BUVEC isolates. Western blotting analyses revealed cyclin E1 to be significantly enhanced in its abundance in *B. besnoiti*–infected BUVEC when compared with non-infected control cells (*p* = 0.00314; Fig. 3). Due to a technical problem in the BUVEC isolate number 5, the measurements from the non-infected and the *B. besnoitia*–infected cells were erased from the statistical analysis. No significant changes were detected for cyclin A2 or cyclin B1, suggesting that neither G2 phase nor mitosis was affected by *B. besnoiti* infections. In contrast to p57-Kip2, we furthermore detected a significant increase of p27-Kip1 abundance in *B. besnoiti*–infected BUVEC (infected vs control cells, *p* < 0,0379; Fig. 3). This regulative molecule is a cyclin-CDK inhibitor blocking cyclin E-CDK2 complex activation. Overall, current expression profiles suggested that *B. besnoiti–*infected cells experienced a stasis at G1-to-S phase transition.

### *B. besnoiti* infections alter neither chromosome condensation nor mitotic spindle formation in BUVEC

**Fig. 4:**
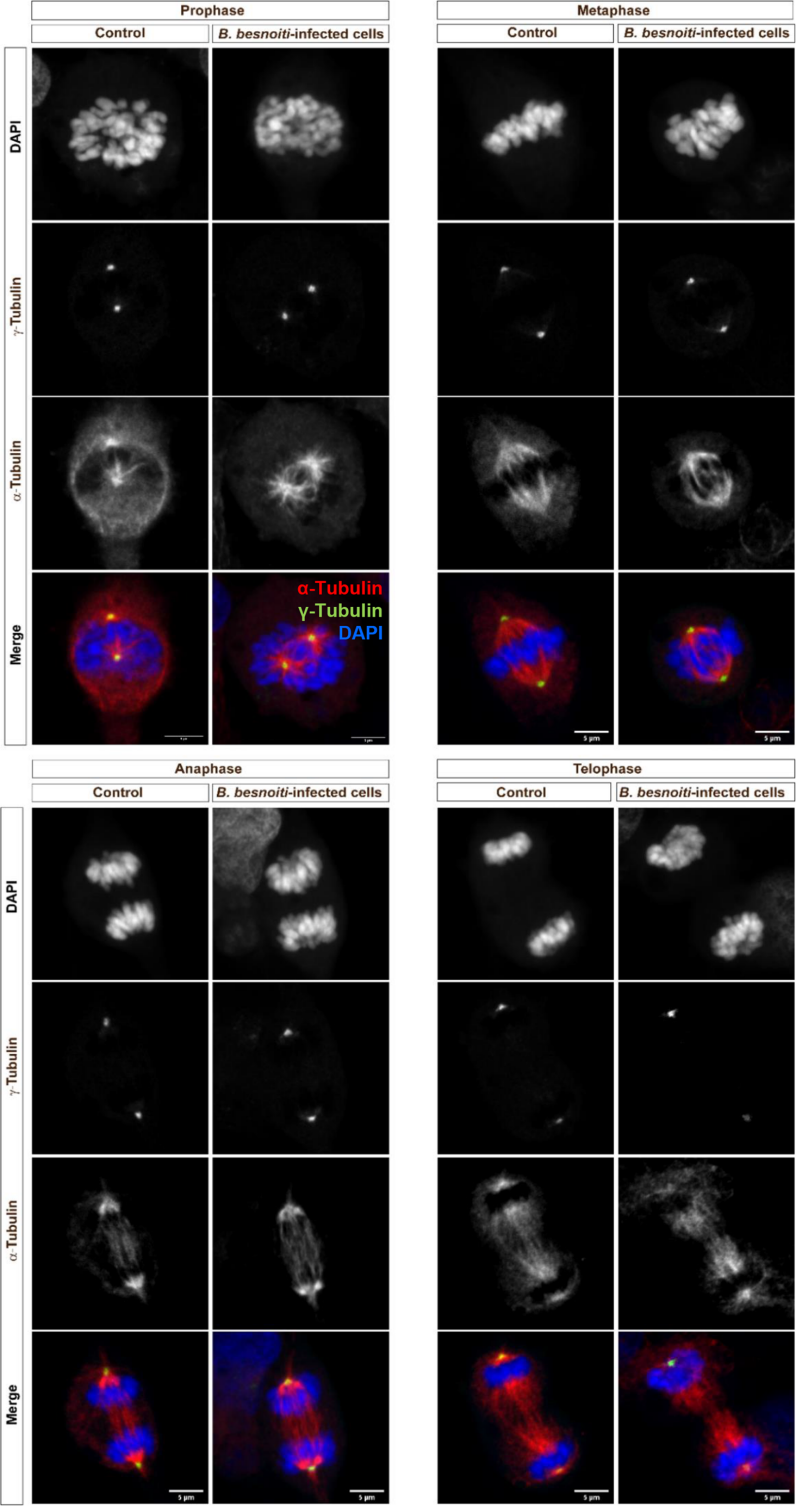
Exemplary illustration of chromosome segregation and mitotic spindle formation in mitotic *B. besnoiti–*infected BUVEC. Mitotic *B. besnoiti–*infected BUVEC and non-infected cells were stained with anti-pHH3 S10 (green) for chromosome detection, with anti-α-Tubulin (red) for mitotic spindle detection. Pictures were taken as a confocal series (z-stack) via confocal microscopy and lately processed as flat images using the ImageJ software (z-projection). The mitotic spindle structure at each cell cycle phase demonstrated that *B. besnoiti* is not intefering with the mitosis process of the host cell. Scale bar represents 5 μm

**Fig. 5:**
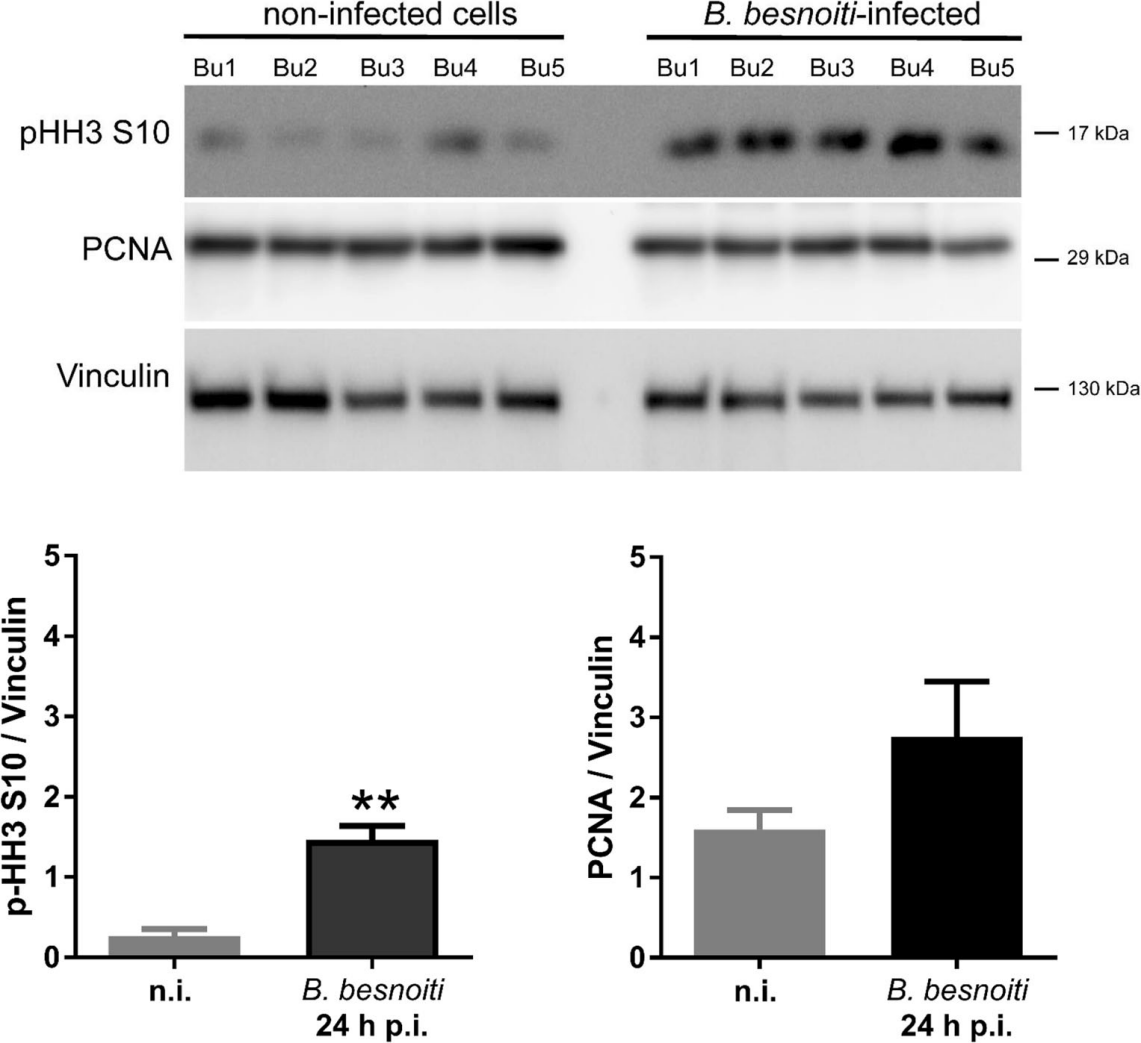
Analysis of phosphorylated-histone H3 and PCNA expression in *B. besnoitia*–infected BUVEC. **a** Five biological replicates of BUVEC were infected with *B. besnoiti* tachyzoites and analyzed by western blotting for phosphorylated histone H3 (S10) and PCNA expression at 24 h p.i. The density of the protein signals was quantified and graphed as a relative ratio to vinculin as housekeeping protein. Both proteins were upregulated, but only p-HH3S10 was statistically significant. Bars represent the median ± SD from five biological replicates. ** = *p* < 0.0741

Since we recently showed that mitosis was impaired on the level of chromosome condensation and mitotic spindle formation in *T. gondii* infections in BUVEC (Velásquez et al. 2019), and since the parasite may modulate host cell cycle on different functional levels, we additionally controlled whether *B. besnoiti* infections may also modulate mitosis progression. Correspondingly, both non-infected and *B. besnoiti*–infected BUVEC were stained for chromosomes (DAPI), microtubules forming the mitotic spindle (γ-tubulin), and for centrosomes (γ-tubulin). In total, 100 cells were analyzed for each condition. Confocal microscopic analyses revealed that *B. besnoiti* infections neither affected chromosome condensation nor mitotic spindle formation during mitosis (Fig. 4). As such, following chromosome arrangement in infected mitotic host cells, we detected normal segregation of chromosomes in prophase, metaphase, anaphase, and telophase (Fig. 4-blue). Likewise, the formation and migration of the tubulin-based cytoskeleton corresponded well to the mitotic spindle shape in control cells for each step (Fig. 4-red). Furthermore, no changes were detected in cases of centrosome formation and localization in mitotic stages (Fig. 4-green). However, when estimating the abundance of phosphorylated histone H3 (Ser10; a classical chromosome condensation marker), a significantly higher expression level of this protein was found in *B. besnoiti*–infected BUVEC when compared with that of control cells (infected vs control cells, *p* < 0,0017; Fig. 5).

### *B. besnoiti* infection induces an increase in nuclear PCNA abundance

**Fig. 6:**
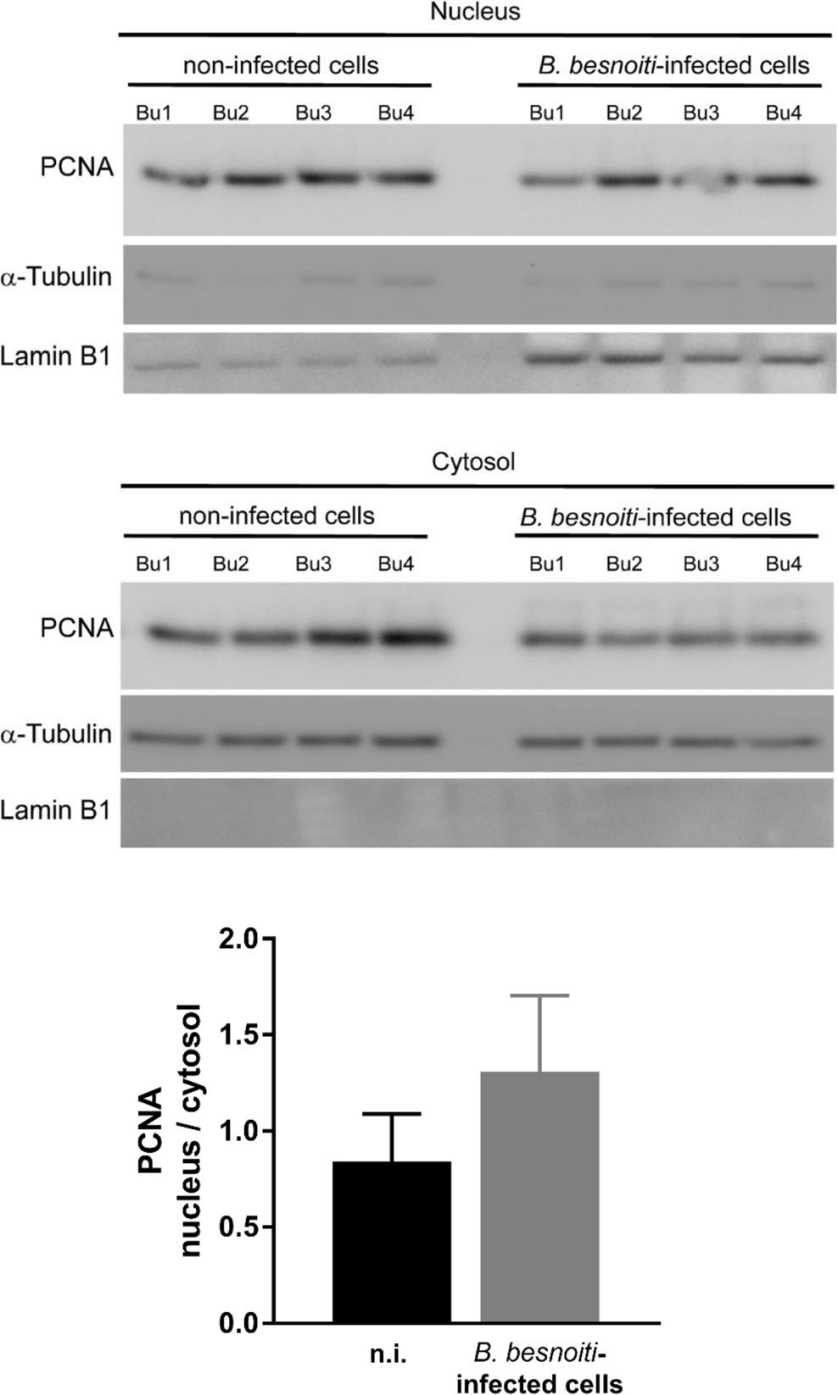
PCNA abundance in nuclear and cytosolic protein extracts of *B. besnoiti–*infected BUVEC. Nuclear and cytosolic protein extracts from four biological replicates of *B. besnoiti–*infected BUVEC and controls cells were subjected to western blotting to quantify PCNA expression. For control of nuclear and cytosolic fraction purity, the abundance of α-tubulin (typical cytosolic protein) and lamin B1 (typical nuclear protein) was analyzed. The density of the protein signals was quantified and graphed as relative ratios: PCNA vs Lamin B1 for nuclear extracts and PCNA vs α-tubulin for cytosolic extracts. The ratio of PCNA/vinculin showed higher values in comparison with the cytosolic one; however, it was not statistically significant. Bars represent the median ± SD from five biological replicates

**Fig. 7:**
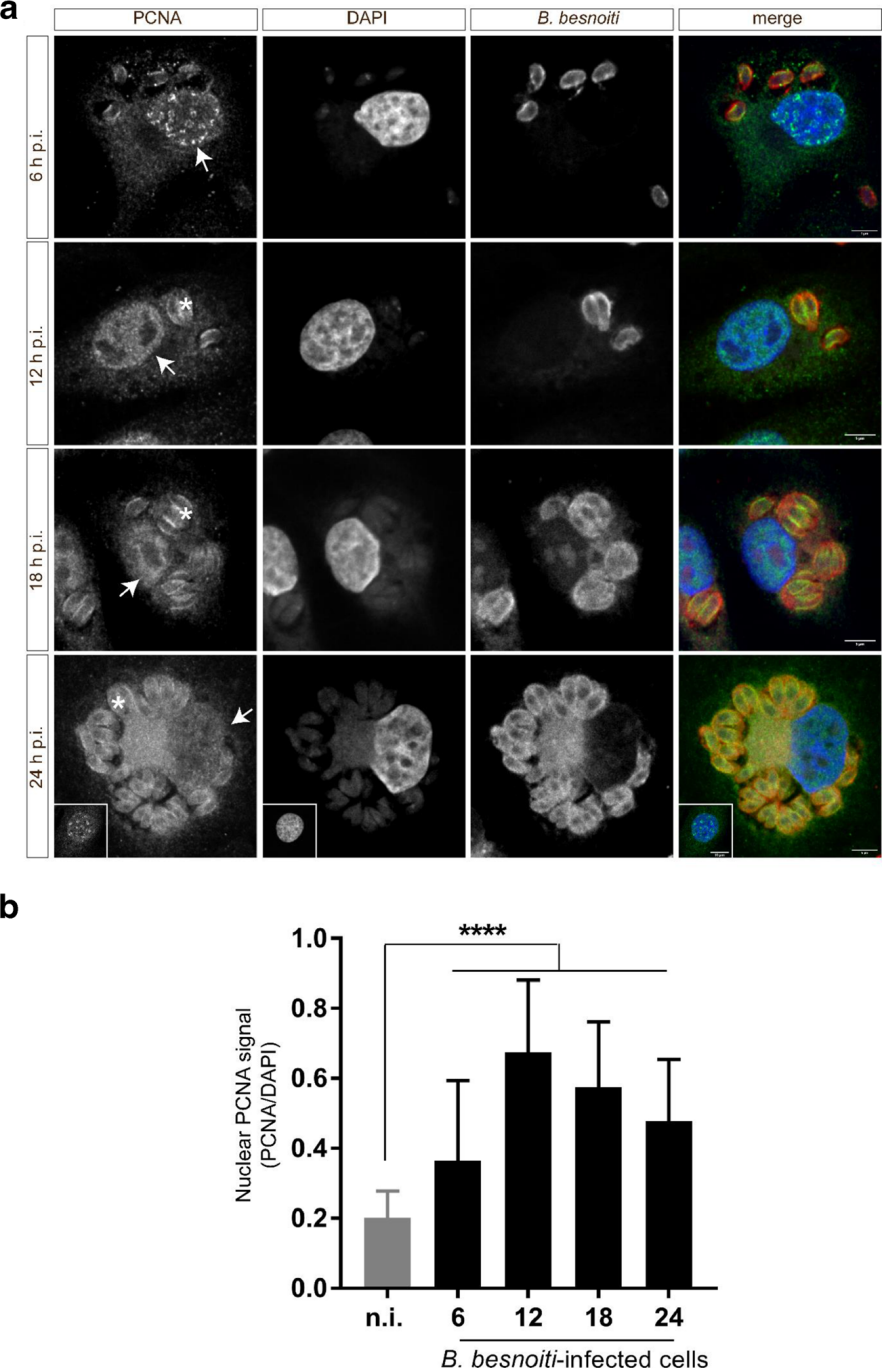
Exemplary illustration of PCNA distribution in *B. besnoiti–*infected BUVEC. **a***B. besnoiti*–infected BUVEC and control cells were stained for DNA (DAPI, blue), *B. besnoiti* tachyzoites (in-house antiserum, red) and for PCNA (green) and analyzed via confocal microscopy. **b** Measurements of nuclear PCNA-related signals in relation to total cell-derived signals during in vitro infection (4–24 h p.i.). The insets in 24 h p.i. pictures represent PCNA localization in the non-infected cells nuclei at the same time. The PCNA signal showed a peak at 12 h p.i., been progressively decreased after 18 h p.i. Scale bar represents 5 μm. Asterisks: surface PCNA localization in *B. besnoiti* tachyzoites. White arrows: PCNA signal in the nuclear compartment. Bars represent the median ± SD against the non-infected cells control. **** = *p* < 0.0001

Proliferating cell nuclear antigen (PCNA) is critically involved in DNA replication and repair, and has cell cycle–dependent properties. PCNA nuclear distribution is characteristic for G phase, early, mid-, and late S phase, and is therefore often used as a marker for sub-stages of DNA replication (Schönenberger et al. 2015). Given that *B. besnoiti*–infected cells seemed to be arrested at G1/S transition, we here analyzed S-phase progression by estimating PCNA localization and nuclear/cytoplasmic abundance. When comparing total PCNA expression in *B. besnoiti*–infected host cells and non-infected BUVEC layers via western blotting at 24 h p.i., no significant differences could be identified, indicating that not total PCNA abundance was not changed (Fig. 5). Given that tachyzoites exclusively reside in the cytoplasmic compartment, parasite-derived signals could falsify these data. We then separated nuclear and cytosolic fractions from both non-infected and *B. besnoiti*–infected host cells (24 h p.i.) for western blot analyses. The purity of each extract was confirmed by the detection of typical cytosolic (α-tubulin) or nuclear (lamin B1) proteins. As expected, PCNA was mainly expressed in the nuclear fraction when compared with cytosolic extracts. In line with our observations described above, the nuclear PCNA abundance in *B. besnoiti*–infected cells was enhanced in nuclear extracts (which are devoid of parasites) (Fig. 6); however, this was not statistically significant (infected vs non-infected cells, *p* = 0.1615). Neither total nor nuclear PCNA abundance showed significant differences when analyzed by WB at 24 h p.i. However, by confocal microscopy, a time-dependent increase of nuclear PCNA abundance with a maximum at 12 h p.i. was observed (Fig. 7). We estimated the nuclear PCNA abundance during 24 h of in vitro infection using immunostaining (Fig. 7) and calculated PCNA signal intensity relative to the host cell nucleus (DAPI signal). In total, 300 cells were analyzed for each condition. *B. besnoiti*–infected cells showed a transient peak at 12 h p.i. (Fig. 7b, infected vs non-infected cells, at all-time points tested, *p* < 0.0001), decreasing after 18 h p.i. The nuclear PCNA localization showed the characteristic pattern for a G1-phase (Fig. 7a).

## Discussion

*B. besnoiti* belongs to the apicomplexan cyst-forming protozoan Sarcocystidae family. During the acute phase of bovine besnoitiosis in vivo, tachyzoites continuously infect host endothelial cells of blood and lymphatic vessels, thereby replicating fast intracellularly (Basson et al. 1970; Bigalke 1981; Muñoz-Caro et al. 2014). In contrast to the well-investigated and closely related parasite *T. gondii*, no data are currently available on the influence of *B. besnoiti* infections on host cellular cell cycle progression even though several reports indicated both species- and cell type–specific reactions in this respect (Maksimov et al. 2016; Taubert et al. 2016). To demonstrate true species-specific reactions and to exclude host cell type–specific effects, we here took advantage of recently published cell cycle–related data on *T. gondii* infections (Velasquez et al. 2019) by using the same in vitro primary host cell system (bovine umbilical vein endothelial cells) under identical culture conditions, thereby standardizing experimental conditions. Using this approach, we here showed that *B. besnoiti* indeed differentially modulates host endothelial cellular cell cycle when compared with *T. gondii* and affects G1/S transition and related molecules in a species-specific manner.

*B. besnoiti* infections affect cattle as intermediate hosts and, as stated above, endothelial host cells of vessels represent the main host cells being infected during acute phase of infection (Basson et al. 1970; Bigalke 1981). In order to be rather close to the in vivo situation and to allow a direct comparison with recently published *T. gondii* data (Velásquez et al. 2019), the current study was performed in a primary bovine endothelial cell type. Furthermore, by using a primary host cell type, false influences on host cellular cell cycle progression or cell division activities driven by cell immortalization or tumoral origin was avoided (Mondello and Chiodi 2013). It is worth noting that we here exclusively analyzed host cell layers for up to 24 h p.i. to avoid false-negative effects triggered by parasite-induced host cell lysis. This contrasts to experimental conditions of other studies on coccidian parasites, such as *T. gondii*, in which host cell proliferation was estimated for up to 48 h p.i. or even 120 h p.i. (Brunet et al. 2008).

By microscopical means, we confirmed that bovine endothelial cells (BUVEC) allowed full intracellular merogony development and resulted in the release of mature and motile *B. besnoiti* tachyzoites. Given that individual primary endothelial cell isolates generally show high inter-donor variability (Joyce 2003; Zhu and Joyce 2004), we here always included at least five BUVEC isolates in each experimental setting. Consequently, we assume that observed differences in host cell modulation in the current study are indeed realistic.

By estimating total BUVEC numbers at 24 h p.i., we showed that *B. besnoiti* infections do not influence host cell proliferation. This finding contrasts with data on *T. gondii* infections which were reported either to dampen host cell proliferation in non-endothelial cell types (Brunet et al. 2008; Molestina et al. 2008; Kim et al. 2016) or to trigger proliferation in the same endothelial cell type we used (Velásquez et al. 2019). Accordingly, we could also not state an infection-driven increase of bi- or multi-nucleated host cells as previously reported for *T. gondii* infections (Velásquez et al. 2019). In this respect, the combination of DRAQ5 staining with live cell 3D-holotomographic microscopy easily allowed nuclear phenotype estimation and nicely illustrated *B. besnoiti* development in BUVEC.

In contrast to *T. gondii*–related cell cycle data (Velásquez et al. 2019), FACS-based quantification of cellular DNA contents did not reveal *B. besnoiti*–driven changes in cell cycle phase patterns when compared with non-infected host cells. This may be based on different reasons: (i) this method is not sensitive enough to detect moderate changes in cell cycle phases, and (ii) when analyzing infected cell layers, this method (which merely estimates crude DNA abundance) cannot distinguish between host cell- and parasite-derived DNA. Given that we achieved a rather high infection rate with several meronts per host cell (2–20, which is typical for endothelial *B. besnoiti* infections in the current experimental set-up), the merge of parasite- and host cell–derived reactions may have masked alterations in host cellular cell cycle phases. Nonetheless, analysis of cell cycle–related molecules involved in regulation revealed cyclin E1 (one of the main regulatory protein of G1-phase and G1-to-S-phase progression) to be selectively upregulated in *B. besnoiti–*infected cell layers since other cyclins (cyclin A2, cyclin B1, or the regulatory molecule p57-kip2) did not show any altered abundance. Cyclin E1 represents an activator of cyclin-dependent kinase (CDK) 2, accumulates at G1/S boundary of cell cycle, and stimulates entry into and progression through S phase (Sauer and Lehner 1995; Ohtsubo et al. 1995; Ekholm and Reed 2000). In somatic mammalian cells, cyclin E1 levels typically decline during S phase, thereby reaching low or undetectable levels by the time replication is completed (Ekholm et al. 2001). Thus, an enhancement or accumulation of cyclin E indicates that *B. besnoiti* infection either triggers cyclin E1 expression or blocks its degradation, which both affect the cellular exit from G1-phase. Another interesting finding was the upregulation of p27-kip1, a CDK inhibitor belonging to the Cip/Kip family. The main regulation of p27-kip1 is mediated by proteolysis via ubiquitin/protease pathway at the G1/S boundary (Lisztwan et al. 1998). This process is closely related with cyclin E1-CDK2 complex activity, which modulates p27-kip1 phosphorylation and allows its entrance into the ubiquitination pathway. Cells can only enter into S-phase when p27-kip1 proteolysis is completed. Thus, the current data suggested that *B. besnoiti* infection affected proper cyclin E1-CDK formation or activation which directly interferes with p27-kip1 proteolysis and is concomitant with G1-phase arrest. The finding of *B. besnoiti*–driven G1/S arrest was revealed as species-specific since *T. gondii* infections triggered stasis of BUVEC in (G2/)M-phase (Velásquez et al. 2019). Furthermore, records on *T. gondii*–driven cell cycle impairment in non-endothelial cells reported an infection-driven shift from G0/G1- to S-phase (Molestina et al. 2008; Lavine and Arrizabalaga 2009) or a host cellular arrest in G2-phase (Brunet et al. 2008), thereby most probably indicating cell type–specific reactions.

G1 phase is followed by S-phase, which signifies the step during which cells duplicate their genome for daughter cell formation. PCNA, which is involved in DNA replication, has cell cycle–dependent properties and typically localizes to nuclear sites of active replication during S-phase (Bravo and Celis 1985; Celis and Celis 1985a; Essers et al. 2005; Sugimoto 2015). Thus, precise PCNA nuclear distribution is used to discriminate sub-stages of S phase (early, mid, and late S phase) (Bravo and Celis, 1985; Celis and Celis 1985a, b; Ersoy et al. 2009). Given that the current data indicated a *B. besnoiti* induced at G1/S transition and assuming that multiple phases may be targeted by the parasite, we here analyzed subcellular PCNA abundance and distribution in *B. besnoiti*–infected BUVEC. One interesting finding, was that host cellular PCNA distribution changed throughout in vitro merogony, showing that even when PCNA was located in the nuclear compartment, it showed a typical pattern of G1-phase after 18 h p.i. (homogeneous distribution in the nucleus). Nevertheless, quantification of nuclear PCNA signals revealed an increase of PCNA abundance during *B. besnoiti* infection in BUVEC peaking at 12 h p.i., which was the last point in time when some S-phase typical pattern could be observed. Correspondingly, when preparing nuclear and cytoplasmic extracts from *B. besnoiti*–infected BUVEC for western blot–derived expression analyses, an increase of PCNA abundance was observed in infected BUVEC. Overall, these data suggest that *B. besnoiti* induces a G1 arrest in a time-dependent manner, thereby hampering host cells to enter into S-phase.

Emerging data on chromosome formation suggest that whenever chromosomes fail to properly replicate in S-phase, subsequent segregation of sister chromatids during mitosis will be impaired as well (Mankouri et al. 2013). For this reason and in order to compare *B. besnoiti*–driven reactions with *T. gondii*–related ones, we here also analyzed host cellular chromosome segregation, i.e., a cellular process that is highly regulated and requires numerous factors for adequate processing, such as molecules mediating DNA movement, DNA linkage to cellular structures, and chromosome maintenance (Duro and Marston 2015). Obviously, proper mitotic spindle formation, being based on tubulin-derived structures for chromosome cell linkage (Inoué and Salmon 1995; Inoué 1997; Rieder and Khodjakov 2003), is also fundamental for adequate chromosome segregation. Recently, we showed that *T. gondii* infection of BUVEC highly interferes with these processes and leads to chromosome missegregation and mitotic spindle impairment (Velásquez et al. 2019). However, *B. besnoiti*–infected BUVEC showed normal chromosome structures and mitotic spindle formation, and no impairment of these processes could be stated, thereby indicating species-specific effects (once) again. Even though the mitotic process did not seem to be affected in *B. besnoiti* infections, analyses on phosphorylated histone H3 (S10) showed a significantly enhanced abundance in *B. besnoiti–*infected host cells. To date, two functions have been assigned to serine 10 phosphorylation of histone H3 with one being related to chromosome condensation and the other being linked to distinct gene transcription activation (Prigent and Dimitrov 2003). In both cases, the serine phosphorylation–mediating kinase appears fundamental (Schmitt et al. 2002; Taylor 1982; DeManno et al. 1999; Cheung et al. 2000; Hans and Dimitrov, 2001). This process selectively occurs in some specific genome regions, depending on the stimuli that the cell received. It has been previously identified that only some subsets of genes are altered; one of these is related with the inflammatory response triggered by cytokines (Saccani et al. 2002). To date, distinct cytokine responses are well-known for *T. gondii* and *P. falciparum* infections (Baker et al. 2008; Fischer et al. 1997), and inflammatory responses were also described for *B. besnoiti* infections in BUVEC, thereby allowing us to hypothesize that an increase of HH3 S10 phosphorylation driven by *B. besnoiti* infection may also be explained by the inflammatory response triggered by the parasite.

In conclusion, we here showed that *B. besnoiti* tachyzoite infection indeed affects host endothelial cell cycle progression in a species-specific manner and avoids host cell cycle progression by a G1-phase arrest. Further research will elucidate the precise mechanisms, signaling pathways, and molecules which are involved in this complex process.
